# Public versus internal conceptions of addiction: An analysis of internal Philip Morris documents

**DOI:** 10.1371/journal.pmed.1002562

**Published:** 2018-05-01

**Authors:** Jesse Elias, Yogi Hale Hendlin, Pamela M. Ling

**Affiliations:** 1 Center for Tobacco Control Research and Education, University of California, San Francisco, San Francisco, California, United States of America; 2 UCSF Department of Medicine, Division of General Internal Medicine, San Francisco, California, United States of America; University of New South Wales, AUSTRALIA

## Abstract

**Background:**

Tobacco addiction is a complex, multicomponent phenomenon stemming from nicotine’s pharmacology and the user’s biology, psychology, sociology, and environment. After decades of public denial, the tobacco industry now agrees with public health authorities that nicotine is addictive. In 2000, Philip Morris became the first major tobacco company to admit nicotine’s addictiveness. Evolving definitions of addiction have historically affected subsequent policymaking. This article examines how Philip Morris internally conceptualized addiction immediately before and after this announcement.

**Methods and findings:**

We analyzed previously secret, internal Philip Morris documents made available as a result of litigation against the tobacco industry. We compared these documents to public company statements and found that Philip Morris’s move from public denial to public affirmation of nicotine’s addictiveness coincided with pressure on the industry from poor public approval ratings, the Master Settlement Agreement (MSA), the United States government’s filing of the Racketeer Influenced and Corrupt Organizations (RICO) suit, and the Institute of Medicine’s (IoM’s) endorsement of potentially reduced risk products. Philip Morris continued to research the causes of addiction through the 2000s in order to create successful potentially reduced exposure products (PREPs). While Philip Morris’s public statements reinforce the idea that nicotine’s pharmacology principally drives smoking addiction, company scientists framed addiction as the result of interconnected biological, social, psychological, and environmental determinants, with nicotine as but one component. Due to the fragmentary nature of the industry document database, we may have missed relevant information that could have affected our analysis.

**Conclusions:**

Philip Morris’s research suggests that tobacco industry activity influences addiction treatment outcomes. Beyond nicotine’s pharmacology, the industry’s continued aggressive advertising, lobbying, and litigation against effective tobacco control policies promotes various nonpharmacological determinants of addiction. To help tobacco users quit, policy makers should increase attention on the social and environmental dimensions of addiction alongside traditional cessation efforts.

## Abstract 2

Using previously secret, internal Philip Morris documents from the early 2000s, Pamela M. Ling and colleagues find that the company's public statements on nicotine as the sole cause of smoking addiction were made in parallel with internal research characterizing addiction's interconnected biological, social, psychological, and environmental determinants.

## Author summary

**Why was this study done?:**

- Tobacco companies publicly denied for decades that nicotine was addictive.
- In 2000, Philip Morris became the first tobacco company to publicly state that nicotine is addictive.
- Today, addiction is understood as a complex phenomenon resulting from both the pharmacology of the addictive substance and the user’s biology, psychology, social milieu, and environment.
- The tobacco industry avidly promotes new nicotine products, emphasizing that nicotine addiction is the key driver of smoking.
- Little is known about how tobacco companies internally understood addiction as they changed their public position.

**What did the researchers do and find?:**

- We examined previously secret internal Philip Morris documents and public company statements and traced Philip Morris’s internal understanding of addiction from the mid-1990s until 2006.
- We found that Philip Morris’s shift from denying to affirming nicotine’s addictiveness was driven by public, regulatory, and legal pressures, not by a substantive change in scientific understanding.
- Philip Morris continued studying addiction through the 2000s to develop successful and potentially safer nicotine products.
- From the mid-1990s to at least 2006, Philip Morris’s internal models of addiction positioned psychological, social, and environmental factors as equally important to nicotine in driving cigarette use.

**What do these findings mean?:**

- Philip Morris’s public embrace of nicotine as the main driver of addiction bolsters the case for tobacco harm reduction but likely does not accurately reflect the company’s internal understanding of addiction.
- Philip Morris has internally understood since at least 2006 that its actions (e.g., advertising, lobbying, and litigation) influence addiction by shaping users’ psychology, social milieu, and environment.
- Philip Morris’s public embrace of nicotine’s addictiveness currently works to redirect policy away from proven social and environmental interventions and toward the promotion of the industry’s potentially reduced harm products.
- To improve addiction outcomes, public health authorities should continue expanding and strengthening social and environmental restrictions on cigarette smoking.

## Introduction

While the tobacco industry has believed since the 1960s that nicotine is addictive [1], it publicly denied this until the early 2000s [2]. Although nicotine is today widely considered addictive [3], this designation is not fixed. The classification of a given substance as addictive depends as much on the contexts in which substances are used as on robust scientific findings regarding these substances’ neurochemical properties [4–8]. Definitions and connotations of “addiction” continually change. Due to this changeability, addiction is best viewed not as a “universal scientific trut[h] to be unveiled or denied” but a “malleable concept situated in specific social, political and scientific contexts” [7].

The tobacco industry’s marketing, lobbying, and litigation against effective tobacco control measures have greatly influenced the public’s understanding of nicotine and smoking [9]. In 1964, when 42% of Americans smoked [10], the surgeon general’s landmark report concluded that smoking was a habit, not an addiction [11]. While the report considered nicotine’s pharmacological properties important, it also framed smoking as the product of psychological drives and social context rather than cigarette pharmacology [5]. To some authors of the report, designating smoking as addictive was incongruent with their view of cigarettes’ role in society. The industry may have further strengthened this perception via an esteemed pharmacologist on the Surgeon General’s Advisory Committee, who maintained undisclosed ties to the tobacco industry [7] and who convinced the committee that the report should employ the World Health Organization’s 1957 definitions of “habituation” and “addiction,” which classified nicotine use as a habit [12].

Through the 1970s and 1980s, mounting scientific evidence and effective media campaigns triggered a large decline in smoking [13]. The resultant shift in the status of smoking—from mainstream to marginalized—corresponded with an emerging understanding of nicotine as an addictive drug [14]. Instead of attributing addiction to the user’s psychology or social context, public health professionals began viewing addiction as a consequence chiefly of nicotine’s pharmacology [15]. In this framework, the US surgeon general designated nicotine as addictive in 1988 [16].

Throughout the 1990s, scientific and popular understanding of nicotine’s addictiveness triggered watershed litigation against the tobacco industry [5, 8]. Internal industry documents and whistle-blower testimony detailed the tobacco companies’ manipulation of nicotine levels, which indicated that the industry considered nicotine an addictive drug [17]. This knowledge motivated the US Food and Drug Administration’s (FDA’s) unsuccessful attempt to regulate cigarettes as drug delivery devices and nicotine as a drug in 1996 [8].

Despite the key role of nicotine in smoking, most addiction researchers and treatment professionals today also designate nonpharmacological stimuli as at least as important as nicotine’s pharmacology [18–22]. The limited efficacy of nicotine replacement therapy (NRT) has shown that smoking is more than a problem of nicotine administration [23–25]. “Cue reactivity” research demonstrates that social and environmental cues associated with smoking—e.g., seeing an advertisement [26–29], seeing an ashtray, drinking a beer, spending time with smoking friends—produce physiological and psychological changes in the user that can trigger substance use [30] and maintain nicotine-seeking behavior, without the administration of nicotine [31–34]. Environmental stimuli associated with smoking can elicit cravings [35, 36], just as stimuli associated with quitting smoking can reinforce cessation behavior [37, 38].

Given the strength of these social and environmental factors, tobacco addiction is best understood not as solely a “brain disease” [39], in which users’ physiological dependence renders pharmacotherapy necessary for quitting, but as a “biopsychosocial” disorder [40], in which the user’s biology (e.g., dopaminergic pathways, brain circuitry), psychology (e.g., coping mechanisms, identity formation), sociology (e.g., relationships, socioeconomic standing), and environment (e.g., access to cigarettes, exposure to advertisements and cues) all interact and overlap with one another and with nicotine’s pharmacology in complex, varying, and mutable ways to determine the user’s smoking addiction and cessation prospects [41].

While addiction research continues to uncover how these components interact, little is known about how tobacco companies internally viewed addiction after the 1998 Master Settlement Agreement (MSA). As tobacco industry research on tobacco has historically been decades ahead of independent science, critically examining industry discussion regarding addiction may provide important context for new tobacco product regulation.

Public health professionals’ and policy makers’ understanding of addiction has historically informed subsequent healthcare practice and policy. Through most of the 1970s, scientists considered smoking a problem of individual willpower, and cessation interventions were consequently limited to psychological interventions [15]. In the early 1980s, however, NRT displayed early promise in clinical trials in assisting cessation [42, 43], leading to interventions targeting nicotine’s pharmacology. The decline of smoking rates in the face of limited real-world NRT success [25], however, suggested that those social and environmental interventions also introduced in the 1980s (e.g. high taxes, smoke-free restrictions, advertising restrictions) also worked to lessen addiction’s tenacity among smokers [44, 45]. While public health professionals agree that combining NRT, behavioral counseling and environmental interventions effectively diminish use [10], opinion is divided as to the role new potentially reduced harm products should play in combatting cigarette use and nicotine addiction [46]. This article analyzes Philip Morris’s (PM’s) internal understanding of addiction immediately before and after publicly admitting nicotine’s addictiveness.

## Methods

This study is based on qualitative analysis of previously secret internal tobacco industry documents available through the Truth (formerly Legacy) Tobacco Industry Document Library (https://industrydocuments.library.ucsf.edu/tobacco/). Documents were retrieved between December 2016 and April 2018. These searches combined traditional qualitative methods of textual analysis with iterative search strategies, analyzing documents chiefly on the basis of relevance to the research question and novelty of information. We excluded documents irrelevant to the analysis on grounds of repeated information, or unverifiable content without supporting documentation.

To assess the internal consistency of documents, we reviewed and organized documents thematically and chronologically to create a time line of events and pertinent changes in PM’s position on addiction and the role of nicotine therein. When document content was ambiguous, we conducted targeted searches based on personnel, project titles, or key words mentioned in that document to further clarify document authorship, content, and/or significance. Per standard archival research methodologies [47, 48], all authors read and reread documents to interpolate meaning and situate the importance within the context of other collected documents.

JE and YH conducted initial broad key word searches, which included “nicotine addiction,” “addiction position,” and “addiction model,” all of which yielded more than 40,000 results. While we initially reviewed documents from other companies, especially R.J. Reynolds, we narrowed our search after a month to focus on PM after identifying PM’s (now Altria and Philip Morris International [PMI]) vanguard shift from denying to embracing nicotine’s addictiveness. Having limited searches to the PM archive, we conducted the same key word searches, which yielded approximately 11,000 results.

After all authors had discussed documents for the initial search period, JE and YH identified a decisive break in PM’s position on addiction following the introduction of MSA litigation. Consequently, we confined our subsequent searches to the two bifurcated eras: 1988–1998 (from the year of the surgeon general’s report on nicotine to the year of the MSA) and from 1998 to the present. Based on our initial search queries, we employed standard snowball sampling techniques [49–51], refining subsequent searches and employing additional Boolean operators in addition to year and publication type filters. While searches in the pre-MSA era yielded several thousand results, several dozen key documents, combined with journalistic coverage of the period, presented a cohesive and in-depth view of PM’s private and public positions.

Further searches focused on PM’s “consensus groups,” which previously collected documents indicated as the locus of PM’s discussions on nicotine addiction and addiction more broadly. Targeted subsequent searches on the PM “Addiction Consensus Group” yielded approximately 700 documents, which discussed the deliberations and findings of three successive groups. We also searched for background information on group members; consensus group report drafts, presentations, and communications; company directives and planning documents; annual company reports; and independent literature cited within internal reports.

We examined documents with adjacent reference (Bates) numbers and dates until we reached saturation, wherein additional searches uncovered only previously seen documents or duplicates of said documents. In excluding duplicates of documents, documents that repeated already ascertained information, documents that either contained irrelevant information or that were unverifiable “restricted documents,”—those protected on grounds of lawyer–client privilege or trade secrets—our iterative process ultimately generated 153 documents on which we based our final analysis.

To further contextualize and verify that the company acted on the information found in the documents, we triangulated this archival research with outside sources to verify and expand our understanding of document contents. Such sources included independent scientific literature, online news engines (e.g., Google News, Lexus Nexus, Access World News), and Altria’s and PMI’s current public statements on addiction and nicotine, made both in presentations by company officials and in statements found on the two companies’ websites (www.altria.com and www.pmi.com). This paper organizes these internal documents and external publications and communications chronologically up to the present day.

## Results

### Philip Morris’s addiction position through the 1990s

Alongside other major tobacco companies, PM long publicly denied the addictiveness of smoking and nicotine. In 1994, PM claimed to the American Drug Abuse Advisory Committee that because the scientific community lacked a consensus on the definition of addiction, a substance’s addictiveness was far more a matter of opinion than verifiable scientific fact [52]. Drawing on more traditional criteria, which judged a substance addictive only if it caused intoxication, dependence (i.e., withdrawal), and tolerance, PM argued that because smoking does not intoxicate, neither smoking nor nicotine should be considered addictive [52].

A 1995 internal slide deck with handwritten revisions entitled “Addiction” shows internal skepticism on the part of PM scientists that nicotine was the exclusive driver of addiction [53], as the FDA had begun arguing. This document, later recycled in a 2000 presentation on addiction [54], claims that “nicotine is but one of the determinants of smoking behavior,” with biological, psychological, and sociological factors also playing major roles [53]. Consequently, quitting smoking was “not simply a matter of replacing the nicotine” [53]. In an internal 1996 document entitled “Statement of Position,” PM noted both the failure of NRT to adequately replace nicotine from cigarettes, as well as the growing number of “chippers” (i.e., social smokers), who consistently smoked but never became addicted [55]. This led the company to internally conclude that “classifying nicotine as addicting may be good public health—[in that it would discourage potential smokers from starting, and give current smokers helpful tools for quitting]—but it does not appear to be good science” [55]. PM later expanded on this position. An internal presentation first circulated in 1997 entitled “Cigarette Smoking & Causation, ETS and ‘Addiction’—The Facts and Positions” [56] was later expanded [57] and circulated among PM senior counsel [58]. Here, PM reaffirmed that the “nicotine addiction hypothesis [i.e., the idea that people smoke exclusively for the nicotine] is much too simple to explain this complex behavior in which nicotine plays a significant, but not exclusive role” [56].

### Philip Morris changes course

Through the 1980s, tobacco companies routinely denied nicotine addiction; as a Tobacco Institute internal memo noted, the claim of nicotine addiction was “the most potent weapon a prosecuting attorney can have,” emphasizing that tobacco companies “can’t defend smoking as ‘free choice’ if the person was ‘addicted’” [59]. In the late-1990s, however, unprecedented legal losses and plummeting public opinion prompted a change of tactics [17, 60]. In an October 1997 presentation to the US Senate Judiciary Committee, PM conceded that nicotine had “mild pharmacological effects, and that, under some definitions cigarette smoking is ‘addictive’”[52], while nonetheless insisting that any definition of addiction must include “historically accepted and objective criteria such as intoxication and physical withdrawal” [52]. Despite its public scientific disagreement with the public health establishment, PM nonetheless pledged to “refrain from debating the issue” outside of litigation so as to “ensure that there is a single, consistent public health message on the issue of addiction”[52].

In 1998, 46 states signed the MSA with PM and the three other major tobacco companies, requiring the industry to pay states US$206 billion over 25 years [61]. Following this reprimand, the largest payment to the public of any industry in history, PM leadership viewed admitting smoking’s addictiveness as beneficial for two reasons. First, executives hoped the settlement would protect the company against future, costly suits from smokers seeking compensation [62]. If PM could claim it had informed smokers of tobacco’s hazards and addictiveness, the company was less likely to be held liable for damages. Second, admitting nicotine’s addictiveness bolstered PM’s legal defense ahead of the US government’s lawsuit against tobacco manufacturers for deceptive practices under the Racketeer Influenced and Corrupt Organizations (RICO) Act. In 1999, several weeks after the US attorney general filed the RICO suit [62], PM updated its website to state that the evidence base overwhelmingly indicated that “cigarette smoking is addictive, as that term is most commonly used today” [56]. Charged, among other counts, with misrepresenting the addictiveness of nicotine, PM argued in court that a finding of liability was only possible under the RICO act if past fraud would likely continue. Because its new website provided some information on the dangers of smoking and nicotine’s addictiveness, PM argued there could be no such future fraud [62]. In 2000, criticism drove PM to further clarify that the company itself agreed with the evidence base designating smoking and nicotine as addictive [62].

While R.J. Reynolds, PM’s biggest US competitor, continued to group nicotine with caffeine and chocolate rather than heroin and cocaine [63], PM grasped the business implications of accepting nicotine as addictive. PM’s admission of nicotine’s addictiveness represented an early component in the company’s strategy of “societal alignment”—appearing responsible to the public in order to rehabilitate the company’s beleaguered public image, stock value, and social influence, and as protection from future lawsuits [64, 65]. To this end, PM spent US$250 million in advertising in an attempt to improve its name and reputation [66]. In November 2001, as part of this rebranding, the company announced that it would change its name from Philip Morris to Altria.

In 2001, the Institute of Medicine’s (IoM, now the National Academy of Medicine) conditionally endorsed tobacco harm reduction [67]. Altria viewed this endorsement—the first by a major public health institution—as a means to both improve its image and bolster falling profits [68]: the company would deepen its investment in reduced-risk products of the sort the report solicited. Two PM addiction researchers, Bruce Davies and Richard Carchman, presented findings to the IoM during the group’s deliberations, in which they argued that different tobacco products should be regulated differently, according to relative harm [69]. Given the unpopularity of potentially reduced exposure products (PREPs) on the market at the time of its release, the report did not initially resonate with the broader public health community. Following the success of electronic cigarettes, however, the IoM report came to inform the industry’s messaging around its alternative nicotine products and embrace of tobacco harm reduction [70].

Having affirmed nicotine’s addictiveness, PM justified expanding its product portfolio in subsequent annual reports to market products claimed to be safer to smokers who could not or would not quit [68, 71]. Altria framed new products as both capable of “reduc[ing] the harm caused by smoking” and as central to the company’s “ability to accelerate growth in the future” [72]. In 2006, the US federal court legally barred tobacco companies from ever again denying nicotine’s addictiveness [73], thereby further pushing PM to pursue reduced harm products.

### Addiction Consensus Group

Despite popular consensus that nicotine, by itself, is an addictive substance, PM’s internal understanding of addiction exhibits a much more complex understanding. Addiction research was reviewed internally by PM’s “Addiction Consensus Group,” first formed in 2000, the “Nicotine Addiction Consensus Group” in 2004, and the “Determinants of Smoking Exposure Consensus Group” in 2005. While each consensus group’s conclusions differed slightly, each group conceptualized addiction as complex and multifaceted, even after physiological dependence set in.

The “Addiction Consensus Group,” first convened in February 2000, sought to better understand why smokers initiated, continued, and ceased smoking [52]. The group was made up of PM pharmacologists and psychologists. The group’s objectives were to clarify.

1.) Current scientific understanding of addiction among PM scientists 2.) How nicotine and/or smoking fit into this understanding 3.) The extent to which nicotine determines smoke exposure and 4.) Thereby to contribute to potential product modifications by the 3^rd^ quarter of 2000 [52].

Following a multidisciplinary literature review, the group characterized addiction as “essentially a multidimensional question” and smoking as a “complex, highly interactive behavior involving psychosocial, sensory and pharmacological elements” [74].

It appears from deliberations that the group considered the term “addiction” to reduce smoking to a crude phenomenon, wherein exposure to a drug automatically provoked biological responses from the user. Because smoking was instead a “complex, highly interactive behavior” involving diverse sciences, the group preferred the term “smoking behavior” [52]. The group reported that nicotine acted “peripherally as well as centrally” but that it was “not the sole determinant of smoking behavior” [74]. The group concluded that while “some people may smoke for central effects of nicotine…scientific evidence for this notion is not compelling” [75]. Other tobacco smoke chemicals’ modulation of the effects of nicotine and “multiple psychological and sociological behaviors” also determined smoking behavior [74].

**Fig 1:**
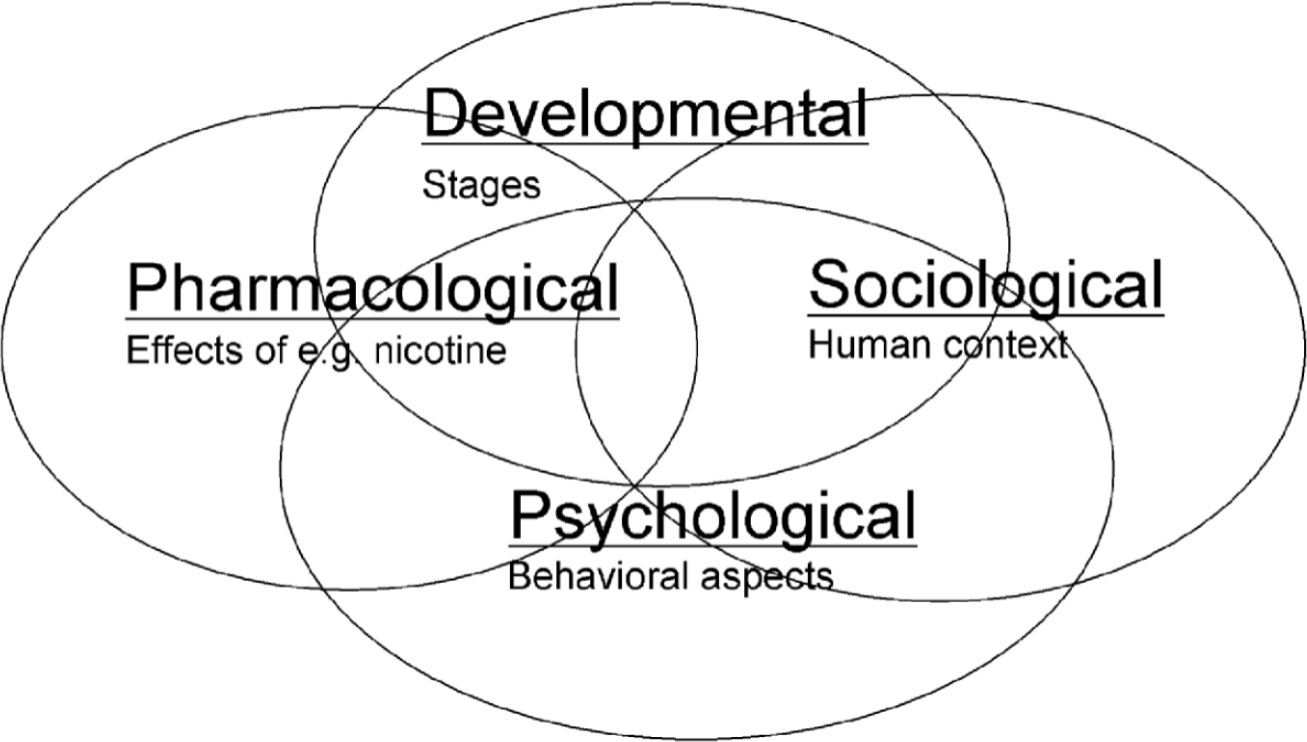
From the “Addiction Consensus Group’s” final report [78]. After a year and a half of deliberation, PM scientists concluded that smokers did not smoke exclusively for nicotine. Rather, addiction was the product of the complicated interactions and reinforcing effects of nicotine’s pharmacology in combination with the user’s psychology, sociology, and the user’s developmental stage of smoking (i.e., initiation, maintenance, cessation, or relapse). PM, Philip Morris.

In addition to nicotine’s pharmacological components (e.g., reinforcing effects, biochemical changes in the brain, withdrawal), the user’s psychology (e.g., stress management skills), sociology (e.g., peer group, socioeconomic and educational levels), and developmental aspects (e.g., whether the smoker was initiating use, maintaining use, quitting, or relapsing) were all equally important in explaining smoking behavior (Fig 1) [52]. This is consistent with epidemiological literature [76]. The group also professed a need for deeper research in each area—always with the aim of developing products “physically and sensorially acceptable to adult smokers” [77] that could “provide reduced risk to smokers, as discussed in the recent IOM report” [52].

### “Chippers” and addiction

The “Addiction Consensus Group” proposed obtaining data on “chippers” so as to better understand how the pharmacological, developmental, sociological, and psychological components of addiction interacted to determine smoking behavior [52]. Public health defines “chippers”—which PM has also referred to as social smokers—as nondaily smokers who smoke five or fewer cigarettes per day, at least four days per week, for at least two years, without developing physiological dependence [79]. Because chippers start and quit smoking at will despite prolonged exposure to nicotine, understanding chipper smoking motivations promised to reveal “the underlying behavioral and non-behavioral mechanisms [of smoking]…[which could] serve as a model for reduction of the number of smoked cigarettes in non-chippers and by that reduce harm in the population” [80].

PM scientists took up this call in a literature review later modified and published as a 2009 article in the journal *Psychopharmacology* [81]. The paper’s authors, all PM scientists, including addiction consensus group member Richard Carchman, estimated that as many as 25% of all smokers were chippers. Among certain groups—such as Latinos in California—chippers constituted as many as 70% of smokers [82]. Citing independent research showing that only half of current smokers met the accepted criteria for addiction under guidelines established by the Diagnostic and Statistical Manual of Mental Disorders, the PM authors argued that repeated cigarette smoking may not necessarily lead to nicotine dependence [82]. Instead, chippers demonstrated that large portions of the population do not smoke for nicotine acquisition but for nonpharmacological reasons. The PM scientists cited one study which framed the “much-debated harm reduction approach…[as] quite inappropriate for this group,” suggesting that social norm change would more effectively attenuate use [83]. The PM report concluded that a significant proportion of chippers used to be regular smokers, indicating “a dynamic process between daily smoking and chipping” [82]. The authors later encouraged PM to further explore the “business implications” of this finding [84].

### Nicotine Addiction Consensus Group

In February 2004, PM began an initiative entitled “determinants of exposure.” As part of this multicomponent project, PM formed the “nicotine addiction consensus group” to determine if nicotine, by itself, was addictive [85]. This five-person group included two former “addiction consensus group” members. Early in its deliberations, the group framed addiction as a “behavioral [i.e. psychological] phenomenon” [85], situating addiction on a spectrum rather than existing dichotomously [85]. Psychosocial, sensory, and pharmacological variables all contributed to the smoker’s repetition and, in some cases, loss of control. Nonetheless, given science’s ever-changing understanding of addiction, PM understood its conceptualization of addiction as “a moving target,” to be updated as the evidence base developed [85].

In the group’s concluding 2005 presentation delivered to PM’s senior team [86], the “nicotine addiction consensus group” concluded that “nicotine per se should have substance dependence potential…[for] between 2–50 percent in that portion of the population using nicotine (i.e. smokers, NRT users)” [87]. Put differently, the consensus group concluded that nicotine was not the most salient aspect driving compulsive use for 50%–98% of tobacco users.

The group also reaffirmed the conclusion of the first addiction consensus group, that smoking is a “complex, highly interactive behavior involving psychosocial, sensory, and pharmacological elements” [87], and agreed with the “overwhelming conclusion of the medical and scientific consensus that smoking is addictive” [87], even if their investigation found that nicotine played a much smaller role in addiction than was popularly believed [87]. Like the previous consensus group, the group foresaw applying these findings to develop PREPs and other “enjoyment products” [87].

### The determinants of smoke exposure consensus group

In 2005, a senior PM research scientist, Candace Adams, presented a “Proposed Model of Adult Smoking Determinants,” framing smoking as a “multi-dimensional, multi-faceted, highly interactive phenomenon involving psychosocial, sensory and pharmacological factors” [88]. Adams also argued that current addiction research focused on “the pharmacological factor at the expense of other factors,” and neglected the “complex nature of adult smoking [and] inter- and intra-individual variation” [88]. Adams’ new model, however, conceived of addiction as the interaction between psychosocial, pharmacological, and sensorial factors [88].

A third consensus group, including two addiction consensus members along with three other scientists, later expanded on this work and examined smoking’s “determinants of exposure” [89]. Through the “determinants of exposure consensus group,” PM hoped to identify which “components of the smoking experience…might impact a smoker’s exposure to cigarette smoke constituents” [90]. The research group closely linked smoking behavior and, by implication, addiction to smoke exposure, stating that determining which compounds in smoke “influence smoking behavior and thus exposure” could facilitate the development of reduced harm products [90].

**Fig 2:**
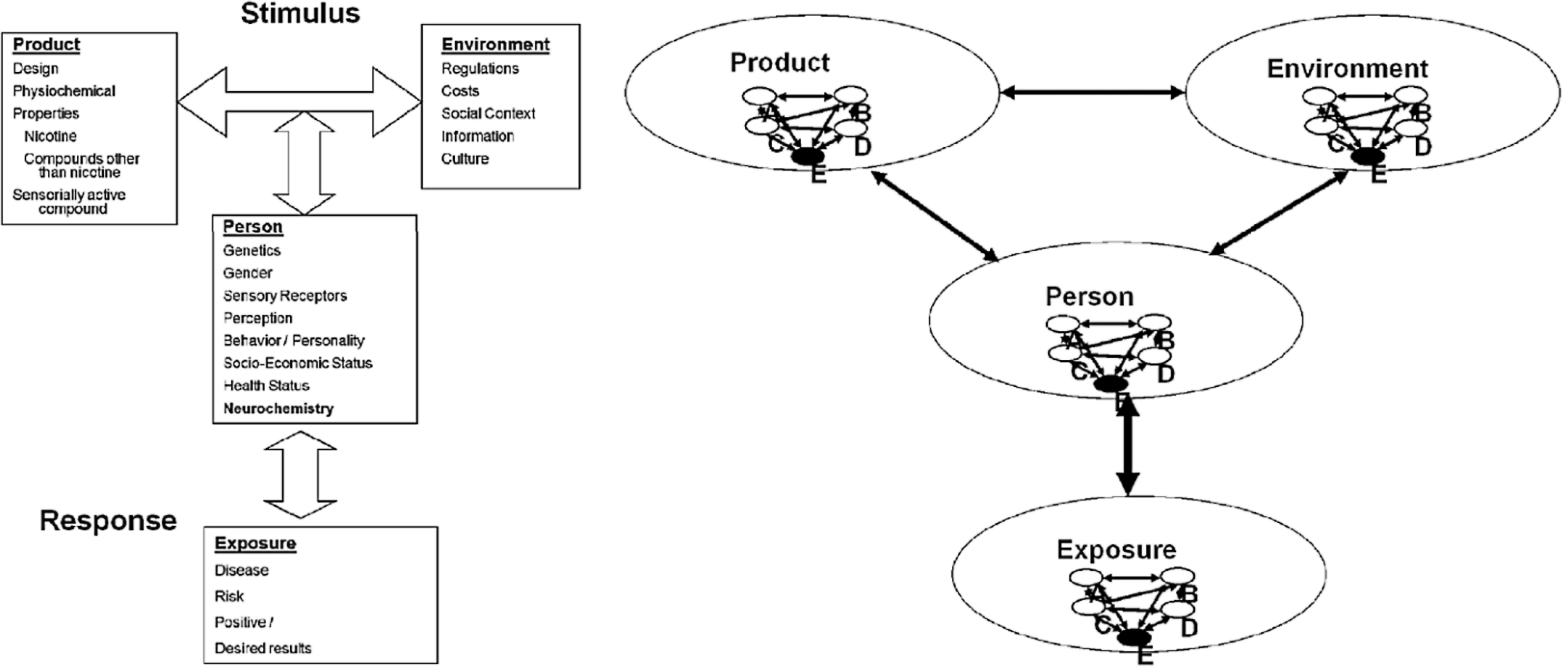
Philip Morris’s 2005 “Determinants of Exposure Consensus Group” diagrams their addiction model in two ways [91]. Left: Stimuli interact to determine response, which then interacts with stimuli. Product: design, physiochemical properties (i.e., nicotine, compounds other than nicotine), and sensorially active compounds. Environment: regulations, costs, social context, information, and culture. Person: genetics, gender, sensory receptors, perception, behavior/personality, socioeconomic status, health status, and neurochemistry. Exposure: disease, risk, and positive/desired results. Right: within each stimulus/response, A, B, C, D, and E interact with each other, affecting the larger component. A, Sensation; B, Perception; C, Genetics; D, Behavior; E, Exposure.

The group’s concluding report presented a new model clarifying how smoking behavior and addiction determined exposure to tobacco smoke [91]. Here, the stimuli motivating use (stimuli designated as “product,” “person,” and “environment”) all interacted to determine the response (i.e., exposure to cigarette toxicants), which in turn influenced the stimuli. Each stimulus and the response itself comprised individual mediating characteristics: “sensation,” “perception,” “genetics,” and “behavior,” which influenced their encompassing characteristic (Fig 2). While it is unclear what the group precisely meant by each of these mediating characteristics, the model nonetheless diagrams smoking behavior (the determinant of exposure to smoke) as a complex, multicomponent phenomenon.

The authors also wrote that environmental factors—such as costs, smoking restrictions, and anti-smoking advertising—helped explain California’s lower smoking prevalence compared to other states, and that “antismoking activities may, in part, be responsible for the dramatic increase in ‘chippers’” [90]. These conclusions implied that by changing smokers’ environment, California had changed smokers’ behavior, reducing the tenacity of addiction in the smoking population [90]. In this model, PM noted the role of nicotine on addiction, while unwittingly indicating the company’s own role in addicting smokers through their determinative role in shaping both the product and the environment, chiefly through efforts to influence aforementioned regulations [92], costs [93], social contexts [94], information [95], and culture [96] to their advantage.

In 2008, Altria spun off PMI as its international company, although the companies continue to share personnel and product development [97]. In contrast to PM’s earlier findings, both companies have embraced the notion of nicotine’s addictiveness to develop future business opportunities, framing nicotine as so addictive that public health institutions must offer reduced harm products for those smokers who cannot or will not quit [98]. In 2016, PMI’s Director of Scientific Engagement stated that “because there are more than a billion smokers worldwide,” PMI must deliver “a variety of different… ‘smoking experiences’ so that smokers [who do not quit] can find something that they can switch to that suits their needs and preferences” [99]. In 2017, PMI’s website stated that “only when a large number of…smokers switch from cigarettes to better products” would a “significant public health benefit” occur [100].

## Discussion

### Key findings

PM and other tobacco companies long publicly denied nicotine’s addictiveness, largely on the grounds that public health institutions employed contrastive definitions of addiction. To improve the company’s image following the MSA and to guard against an adverse RICO ruling, however, PM became the first tobacco company to publicly admit that nicotine was addictive. This admission aligned with public health’s perception of nicotine as the principal driver of cigarette use. In comparing PM’s public and internal framings of addiction, however, one finds a discrepancy: from the mid-1990s through at least 2006, PM internally understood addiction as the sum of complex interactions between nicotine’s pharmacology and the user’s biology, psychology, social milieu, and environment.

Today, Altria and PMI publicly portray tobacco product substitution alone as a sufficient public health intervention. This analysis of internal documents suggests, however, that such a policy is likely inadequate so long as other social and environmental tobacco-related cues, (e.g., public smoking and tobacco advertising) remain in place. The excluded element in PM’s internal analyses, implicit in PM’s own understanding of the social and environmental determinants of addiction, is the tobacco industry itself: the vector of tobacco addiction that foments and maintains addiction not just through its provision and manipulation of nicotine but through its active influence on smokers’ psychology, social milieu, and environment.

### What this paper adds

Despite PM’s apparent change-of-heart, this analysis suggests that the company’s current public framing of addiction is—at least as of 2006—as opportunistic as its initial denial of nicotine’s addictiveness. In reducing addiction treatment to exchanging “dirty” nicotine (i.e., from a cigarette) with “clean” (i.e., noncombustible), PMI obviates the need for comprehensive public health interventions in favor of increasing the number of “choices” available to individuals, thereby replacing the known harm of cigarettes with the unknown harms of new nicotine products. This emphasis on improved nicotine administration aligns with the brain disease model of addiction, which holds that because repeated exposure to a substance induces physiological changes in the user, most users will require pharmacotherapy to quit [39]. Most major funding bodies and many public health advocates subscribe to this view [101, 102]. This model is an important and welcome departure from past models that blamed smokers for lacking moral fiber or willpower necessary to quit. The biopsychosocial model, however, similarly exonerates smokers of blame for their addiction, albeit on a different basis: instead of attributing dependence solely to nicotine’s pharmacology, the model encourages public health to recognize those psychological, social, and environmental cues that also drive addiction, which pharmacotherapy cannot address and which industry efforts reinforce. The evidence base to date and PM’s internal understanding of addiction suggest the biopsychosocial framework more accurately represents addiction and is more likely to generate policy that improves health outcomes.

### Study strengths and limitations

The fragmented and disorganized nature of the tobacco industry documents archive means we may have missed documents relevant to our analysis. Relevant information may also be present within the archive’s many restricted documents, all of which are inaccessible on the grounds that information therein constitutes privileged legal communications. Tobacco companies often use this designation to avoid making internal documents public [103, 104]. Another weakness of the archive is that documents are not up to date—we found documents detailing comprehensive company efforts to understand addiction only until 2006. PM companies (now Altria and PMI) may have changed their internal stance on addiction since then. Nonetheless, we believe this is unlikely: PM scientists consistently understood addiction as a multifactorial phenomenon, in which nonpharmacological components are at least as important as nicotine in driving use. This is a novel discovery that leverages the unique insights of private company documents.

### Policy implications

By publicly emphasizing nicotine’s innately addictive pharmacology, tobacco companies shift policy focus away from proven social and environmental interventions and toward the adoption of the industry’s new nicotine products [98, 105]. Because, however, nicotine is understood as but one factor driving addiction, policy makers should continue promoting effective nonpharmacological interventions. When social and environmental regulations are strong—e.g., when advertising is banned, plain packaging or graphic warning labels instituted, tobacco taxes high, smoke-free restrictions widespread, and effective tobacco control media campaigns far-reaching—addiction’s tenacity diminishes and smoking prevalence declines [106]. Understandably, then, Altria and PMI’s public communications continuously seek to shift popular understandings of addiction away from its social and environmental determinants (which, if addressed, threaten company profit) toward a simplistic problem of nicotine administration (which stands to protect profit) [98, 105].

Altria and PMI have actively sought government certification of their products as acceptable long-term nicotine maintenance products [107–109]. Additionally, in 2017, PMI announced the establishment of a US$1 billion foundation dedicated to partnering with public health and promoting PMI’s portfolio of reduced harm products [105]. Public health professionals have argued that if PMI was sincerely interested reducing tobacco-related harm, the company would cease sale of cigarettes or, at a minimum, cease actively undermining the World Health Organization’s Framework Convention on Tobacco Control (FCTC) [110, 111].

In July 2017, the FDA began contemplating lowering the nicotine content in cigarettes to below addictive levels and simultaneously making noncombustible sources of nicotine more widely available [112]. While nicotine reductions would greatly help those smokers with strong physiological dependence on nicotine, PM’s own research shows that interventions must simultaneously address the industry-driven nonpharmacological stimuli driving addiction and use. As long as these nonpharmacological drivers remain in place, many smokers are likely to continue using cigarettes despite the availability of reduced harm products.

To comprehensively address addiction, public health practitioners must not let the industry reduce addiction to a problem of nicotine administration, best treated with policy that broadens choice for reduced-risk products and/or pharmacotherapies [113]. Given the lack of long-term data on new potentially reduced harm products’ safety, population health effects, and cessation efficacy [46, 114, 115], as well as the industry’s track record of using partnerships and “safer” products to increase profits rather than genuinely improve public health [73], governments should heed the FCTC, which forbids signatories from any collaboration with the industry [116, 117].

## Conclusion

Our analysis suggests that PM’s (now Altria’s and PMI’s) shift from denying to embracing nicotine’s addictiveness is an opportunistic attempt to maintain future profit and capitalize on tobacco harm reduction. As PM’s internal research indicates, positive health outcomes are more likely to be achieved by complementing NRT and behavioral counseling with ever-stronger environmental interventions addressing the psychological, social, and environmental components of addiction. To date, these broader policies have prevented and treated addiction and disease more effectively than individualized solutions, including pharmacotherapy [13, 24, 25, 40]. Such interventions are especially important given smoking’s increasing concentration among the most marginalized members of society, who are least likely to have access to pharmacotherapy, potentially reduced harm products, or effective cessation assistance services [118–120]. A biopsychosocial model recognizing the broader, industry-driven determinants of tobacco use is more likely to support a comprehensive approach to address tobacco disparities.

## Abbreviations

FCTC: Framework Convention on Tobacco Control
FDA: US Food and Drug Administration
IoM: Institute of Medicine
MSA: Master Settlement Agreement
NRT: nicotine replacement therapy
PM: Philip Morris
PMI: Philip Morris International
PREP: potentially reduced exposure product
RICO: Racketeer Influenced and Corrupt Organizations
